# Immunogenicity of Adalimumab in Bacterial Molecular Mimicry: In Silico Analysis

**DOI:** 10.2196/83872

**Published:** 2025-12-08

**Authors:** Diana Isabel Pachón-Suárez, Germán Mejía-Salgado, Oscar Correa, Andrés Sánchez, Marlon Munera, Alejandra de-la-Torre

**Affiliations:** 1 Doctorate in Clinical Research, Escuela de Medicina y Ciencias de la Salud, Universidad del Rosario, Bogota, Colombia; 2 Neuroscience (NEUROS) Research Group, Neurovitae Research Center, Institute of Translational Medicine (IMT), Universidad del Rosario, Cra 24 # 63C- 69, Bogota, 110911, Colombia, +57 601 2970200 ext. 3320; 3 Pulmonology and Immunology in Pediatrics Research Group, Department of Pediatrics, School of Medicine, Universidad Nacional de Colombia, Bogota, Colombia; 4 Faculty of Health, Medical Research Group (GINUMED), Corporación Universitaria Rafael Nuñez, Cartagena, Colombia

**Keywords:** adalimumab, antidrug antibody, immunogenicity, in silico analysis, molecular mimicry

## Abstract

**Background:**

Adalimumab, a monoclonal antibody targeting tumor necrosis factor α, treats autoimmune diseases but induces antidrug antibodies in 30% to 60% of patients, reducing its efficacy.

**Objective:**

This study aims to investigate molecular mimicry as a mechanism behind this immunogenicity, where bacterial immunoglobulin domains structurally resemble adalimumab’s light chain, triggering immune responses.

**Methods:**

Using PSI-BLASTp (National Center for Biotechnology Information) and PRALINE (Center for Integrative Bioinformatics), there are 40 bacterial antigens homologous to adalimumab, with 8 clinically relevant strains.

**Results:**

Structural analysis revealed 94% amino acid identity between the immunoglobulin domain of *Escherichia coli* strain B1 and adalimumab’s light chain, and 89.67% similarity with *Corynebacterium pyruviciproducens*. Root mean square deviation values confirmed strong structural homology. Additionally, 5 cross-reactive B-cell epitopes were predicted, suggesting overlapping surfaces that may promote immune cross-reactivity and antidrug antibody development.

**Conclusions:**

This study represents a first step toward identifying a potential microbial factor driving antiadalimumab antibody formation. The predicted cross-reactive regions provide specific candidates for further in vitro validation to confirm molecular mimicry and refine epitope mapping. Understanding these mechanisms may ultimately inform the design of less immunogenic biologics and guide clinical strategies to predict and prevent antidrug antibody formation.

## Introduction

Adalimumab is a fully human monoclonal antibody targeting tumor necrosis factor-α, a protein involved in inflammation in various chronic autoimmune conditions [1]. The Food and Drug Administration has approved adalimumab to treat several diseases: rheumatoid arthritis, ankylosing spondylitis, Crohn disease, ulcerative colitis, hidradenitis suppurativa, juvenile idiopathic arthritis, plaque psoriasis, psoriatic arthritis, and uveitis [1]. Despite the humanization of adalimumab, the amino acid sequences of both the heavy and light variable chains near the epitope binding regions within the complementarity-determining regions tend to elicit a robust immune response [1-3]. Immune complexes formed by adalimumab and antigens can reach 4000 kDa, and despite being humanized, they may still be recognized as foreign, triggering antidrug antibodies (ADAs) that reduce its effectiveness [1,2].

Approximately 30% to 60% of patients on treatment with adalimumab eventually experience a reduction in the effectiveness of the treatment [4]. This waning efficacy is believed to be due, in part, to immunogenicity, which refers to the body’s generation of antibodies targeting the biological drug [4]. One potential mechanism underlying this immune response is immune cross-reactivity, where the immune system interacts with structurally similar antigens from different sources. Among these, molecular mimicry—where bacterial proteins share structural homology with therapeutic antibodies like adalimumab—has emerged as a plausible explanation for this phenomenon [5,6]. Four requirements must be met for an infection to be implicated in the molecular mimicry-based development of an autoimmune response: there must be epidemiological data about exposure to the environmental agent and the development of autoimmunity, structural homology between human antigens (or medication) and pathogens, autoantibodies or autoreactive lymphocytes against both human and pathogen antigens, and in vivo evidence in animal models [2,7].

Immunoglobulin domains, widely recognized for their role in the structure and function of key immunological proteins, are highly conserved units across evolution [8]. Interestingly, these domains are not confined to the immune systems of higher organisms but are also present in bacterial proteins, including those of *Escherichia coli* and other enterobacteria [8,9]. Immunoglobulin-like domains are frequently found in these microorganisms in cell surface proteins and fimbrial organelles, where they play essential roles in host cell adhesion and invasion by pathogenic strains. They serve as structural components of pilus and nonpilus fimbrial systems and are members of the intimin or invasin family of outer membrane adhesins [8]. This dual functionality underscores their significance as a possible evolutionary mechanism through which pathogens leverage these conserved structures to evade or modulate the host immune response, facilitating infection and colonization [8].

The immune cross-reactivity highlights the need for a comprehensive understanding of the immune responses induced by microorganisms in drugs. It has important implications for the development and use of pharmacological therapies. Currently, there is no evidence of immune cross-reactivity between adalimumab and microbial antigens. However, cross-reactive immune responses have been reported with other medications and vaccines, leading to thrombocytopenia and autoimmune diseases. Furthermore, patients with underlying autoimmune conditions who develop infections are more likely to produce anti-drug antibodies [5,10-15].

Molecular mimicry has traditionally been investigated in the context of autoimmune diseases and vaccine responses, where microbial antigens resemble host proteins. However, its potential role in the immunogenicity of therapeutic antibodies remains largely unexplored. In this study, we apply this concept to examine whether infections could trigger cross-reactive immune responses toward biological drugs. Specifically, we investigate possible cross-immunogenicity between the immunoglobulin domain of clinically relevant Gram-positive and Gram-negative bacteria and adalimumab using in silico approaches. To our knowledge, this is the first study to propose a mechanistic link between bacterial antigens and adalimumab immunogenicity supported by sequence and structural evidence. By addressing this unexplored aspect of biologic drug immunogenicity, our work provides a novel conceptual framework that may guide future experimental validation and inform strategies to improve therapeutic antibody safety.

## Methods

### Study Design

A workflow image of the method is shown in Figure S1 in Multimedia Appendix 1.

### Sequences Analysis

Adalimumab’s amino acid sequence, identified by its DrugBank Accession Number (DB00051), was obtained from DrugBank [16,17]. The complete amino acid sequences of both the heavy (α) and light (β) chains corresponding to the Fab region were used for the analyses. These sequences include the variable and constant domains, ensuring full structural representation of the monoclonal antibody during alignment and comparison. Adalimumab sequence served as the input for a PSI-BLASTp (version 2.16.0; National Center for Biotechnology Information) search targeting bacterial homologs, using the identifier Bacteria (taxid:2). The length of matched subsequences in PSI-BLASTp is influenced by statistical significance thresholds and the iterative nature of the algorithm, which together help ensure that only meaningful alignments are included in the analysis [18]. Default settings were applied for the general search parameters. For subsequent analyses, amino acid sequences from bacteria of clinical significance to humans were selected [19].

Antigens demonstrating a similarity of ≥30% were considered for further investigation. The amino acid sequences from the chosen microorganisms were aligned with the adalimumab light and heavy chains to ascertain identity levels and pinpoint conserved regions. The PRALINE tool (version 2; Center for Integrative Bioinformatics) [20] facilitated the alignments by identifying regions of similarity, which may indicate functional, structural, or evolutionary relationships among the proteins being compared. The alignment parameters were configured to use BLOSUM62 as the exchange matrix using default parameters unless otherwise specified. Specifically, we used BLOSUM62 as the substitution matrix with a gap opening penalty of 11 and a gap extension penalty of 1 (standard for PSI-BLAST alignments). For the PSI-BLASTp search, 3 iterations were performed with an E-value threshold of 0.001 to enhance sensitivity and identify distant homologs [21]. E-value represents the number of random matches you would expect to find with a score equal to or better than the one observed [22]. Similarly, antigens with a similarity of ≥30% were advanced for further analysis. Also, a high-resolution Protein Data Bank (PDB) file (ID: 3wd5) was sourced from the PDB [23,24], enabling structural analysis.

### Modeling Based on Homology

3D models of selected antigens, for which no reports exist in the PDB, were constructed based on homology using the SWISS-MODEL server (ProMod3) [25,26]. These initial models underwent further refinement with UCSF Chimera (version 1.1.3) [27].

Antigens with experimentally resolved 3D structures were sourced directly from the PDB. The visualization of all models was achieved using PyMOL (version 3.0; Schrödinger, Inc) [25]. Structural homology assessments were conducted using the Ramachandran charts, the Quantitative Model Energy Analysis index, the RMSD metric, and Global Model Quality Estimate (GMQE) values were assessed for all models. RMSD focuses on precise structural alignment, while GMQE provides a broader model quality evaluation. Using both metrics allows researchers to make informed decisions about the reliability and applicability of their homology models in biological research [26,28].

### B-Cell Epitope Prediction

The prediction of B-cell epitopes was carried out using the ElliPro server (version 3.0; IEDB Analysis Resource), using the default parameters that are a minimum score protrusion index (PI) threshold of 0.5 and a maximum distance of 6 Å between residue centers for defining discontinuous epitopes. ElliPro identified linear and discontinuous epitopes based on the protein’s 3D structure (PDB ID: 3WD5) using the PI of residues [29,30]. B-cell epitope prediction methods, such as those using the Ellipro server, generally achieve accuracy rates ranging from 65% to over 70%. Still, ongoing validation and refinement are necessary due to variability in sensitivity and specificity across different prediction tools [27]. Furthermore, epitopes previously identified for adalimumab were retrieved from the Immune Epitope Database (IEDB). This step was essential to explore the potential molecular mimicry between antigens from bacteria and those associated with adalimumab. Epitopes conserved between adalimumab and its bacterial homologs were visualized on the 3D model of the monoclonal antibody using PyMOL version 3.0.

### Major Histocompatibility Complex Class II–Dependent T-Cell Epitope Prediction

T-cell epitope prediction was performed using the NetMHCIIpan version 4.1 web server (IEDB Analysis Resource) [31]. This platform uses an ensemble of deep neural networks trained on large quantitative binding datasets (IC₅₀ values) derived from multiple human leukocyte antigen (HLA) class II molecules, enabling panallelic prediction of peptide-HLA interactions.

The FASTA sequences of the VH and VL chains were analyzed separately. The alleles HLA-DRB1*01:01, HLA-DQA1*01:01/DQB1*05:01, HLA-DPA1*01:03/DPB1*02:01, and HLA-DQA1*05:01/DQB1*02:01 were selected due to their frequency and immunogenetic relevance in diverse human populations commonly used in therapeutic immunogenicity assessments. Epitope scanning was performed with a 15-amino-acid window (15-mer) sliding by one residue to ensure maximal coverage of overlapping peptides. The software generated an affinity score (nM) and a percentile rank for each predicted peptide. Following the server’s guidelines, peptides with rank ≤2.0% were classified as strong binders, while those with 2.0%<rank≤10% were considered weak binders.

### Filtering, Ranking, and Epitope Selection

All prediction outputs were exported in CSV format. The data were subsequently filtered to retain only peptides with an affinity score ≥0.2 and low percentile rank, indicative of stable peptide–major histocompatibility complex (MHC) II complexes. Scores and ranks were compared across both antibody chains to identify high-affinity regions and potential immunogenic hotspots.

### Population Coverage Analysis

To assess the global relevance and potential immunological reach of the predicted MHC class II–restricted T-cell epitopes, a population coverage (PC) analysis was conducted using the population coverage tool available at the IEDB [32].

This analysis estimates the fraction of individuals within defined human populations that are likely to present one or more of the predicted epitopes, based on the distribution frequency of HLA alleles. The tool integrates the predicted epitope–HLA binding data obtained from NetMHCIIpan 4.1 with HLA genotypic frequencies derived from the Allele Frequency Net Database, which compiles large-scale datasets from diverse ethnic and geographical groups worldwide.

All epitopes predicted as strong or weak binders (rank ≤10%) across the selected HLA-DR, HLA-DP, and HLA-DQ alleles were included as input. The analysis was performed for multiple population sets, including global coverage, South American, and European cohorts, representing regions with significant therapeutic use of adalimumab and diverse HLA genetic backgrounds.

The algorithm computes several parameters: projected PC (%), representing the cumulative percentage of individuals expected to respond to at least one of the selected epitopes. Average number of epitope–HLA combinations recognized per individual, reflecting immune response redundancy. PC90 value (PC 90%), indicating the minimal number of epitope–HLA combinations required to cover 90% of the target population.

The results were exported in CSV format and visualized as bar and cumulative distribution plots to illustrate interregional variability in potential T-cell responsiveness. This step provides an estimate of the breadth and universality of the predicted epitope set, allowing prioritization of epitopes with the highest immunological representativeness across human populations.

### Conservation Analysis of Immunoglobulin Domain in Bacteria

The conservation of amino acid residues from the immunoglobulin domain across various bacterial species in relation to adalimumab was analyzed using the Rate4Site algorithm on the ConSurf server (version 1.00) [33]. This algorithm calculates position-specific evolutionary rates using an empirical Bayesian approach. The rates are normalized and categorized into 9 grades, with highly conserved residues assigned a score of 9 and highly variable residues receiving a score of 1. The thresholds for these categories are based on the normalized evolutionary rates calculated by Rate4Site. Residues with scores of 1‐3 are considered highly variable, reflecting higher evolutionary rates and frequent mutations or substitutions across species. Conversely, residues with scores of 7 to 9 are classified as highly conserved, indicating minimal variability and strong evolutionary pressure to maintain their structure and function across species. These conservation rates were then visualized using the structural model of adalimumab obtained from the PDB using the Chimera tool [27].

### Allergenicity Prediction Using AllerTOP

The potential allergenicity of the predicted T-cell epitopes derived from the light chain of the adalimumab antibody was evaluated using the AllerTOP v.2.0 server [34,35]. This bioinformatics tool applies an alignment-independent approach based on auto- and cross-covariance transformation of protein sequences into uniform-length vectors, followed by machine learning classification using a k-nearest neighbor algorithm trained on a curated dataset of known allergens and nonallergens.

All epitopes predicted by NetMHCIIpan 4.1 as MHC class II binders (rank ≤10%) were used as input sequences in FASTA format. Each peptide was analyzed individually to determine its probability of being classified as “Probable Allergen” or “Probable Non-Allergen,” according to the physicochemical descriptors of amino acid residues (hydrophobicity, size, flexibility, and secondary structure propensity).

The results were automatically compared to the training dataset of AllerTOP, which includes more than 2400 allergenic and 2400 nonallergenic proteins, allowing for an indirect homology-free prediction of allergenic potential. The outcomes were exported and tabulated, recording for each peptide (1) the most similar protein identified, (2) the source organism, and (3) the allergenicity classification.

Peptides classified as “Probable Allergen” were further analyzed for potential molecular mimicry with known allergens of plant, fungal, or arthropod origin to assess possible cross-reactivity risks. The combined use of NetMHCIIpan 4.1 and AllerTOP v.2.0 allowed an integrated evaluation of both T-cell immunogenicity and allergenicity potential for the light chain–derived epitopes of adalimumab.

### Ethical Considerations

This study was conducted entirely using in silico methods and publicly available, deidentified data. No human participants, personal information, or identifiable records were accessed; therefore, issues of privacy and confidentiality do not apply. No compensation was provided, as no participants, either human or animal subjects, were involved. Consequently, institutional ethical approval and informed consent were not required for this research.

## Results

### PSI-BLASTp

To explore potential molecular similarities between the monoclonal antibody and bacterial proteins, a PSI-BLASTp search was performed. This analysis was conducted to identify possible cross-reactive epitopes that could contribute to off-target interactions or immunological cross-reactivity, which are relevant for understanding antibody specificity and safety. The search revealed 40 significant matches between the monoclonal sequence and bacterial antigens. From these, 8 sequences corresponding to bacteria of clinical relevance were selected for further analysis (Table 1). Comparative alignment between the adalimumab light chain and the identified bacterial homologs demonstrated an average amino acid identity of 64%. This identity represents amino acid residues that are identical and located in the same position when the sequences are aligned. Higher sequence similarity increases the likelihood of cross-reactivity, as it suggests that the compared molecules may share structurally conserved epitopes capable of being recognized by the same antibodies. The most conserved region was located between residues 74 and 150 (Multimedia Appendix 2), suggesting a potential structural or functional similarity in this segment. The analysis revealed that adalimumab light chain shares sequence homology with a bacterial protein containing an immunoglobulin domain, suggesting possible evolutionary or conformational parallels. Similar results were obtained for the heavy chain, although some alignments involved hypothetical bacterial proteins. Consequently, subsequent analyses focused on the heavy chain and the homologs that could be fully annotated (Table S1 in Multimedia Appendix 3).

**Table 1.** PSI-BLASTp results. This table presents the root mean square deviation (RMSD) values, which measure the average structural deviation between aligned molecules.

Bacteria	Antigen	Similarity (%)	Cod genbank	RMSD^a^
*Escherichia coli*	IDCP^b^	94	HEC3531043·1	0.2
*Corynebacterium pyruviciproducens*	IDCP	89.67	WP_280195946·1	0.35
*Klebsiella pneumoniae*	IDCP	82.86	WP_317090695·1	0.3
*Vibrio vulnificus*	IDCP	75.00	MCA0777086·1	0.5
*Pseudomonas* sp.	IDCP	67.80	MCX2891938·1	0.6
*Helicobacter pylori*	IDCP	62.84	WP_304481324.1	0.5
*Salmonella enterica*	IDCP	55.56	MBS2599521.1	0.9
*Staphylococcus aureus*	IDCP	43.84	WP_282719268.1	0.8

a RMSD values below 1 Å indicate an exceptionally high degree of structural similarity, often reflecting near-identical alignments. This level of similarity is particularly relevant in studies of molecular mimicry, as it suggests that the structures may share conserved functional or antigenic regions, increasing the likelihood of cross-reactivity. Such low RMSD values underscore the robustness of the alignments and the potential biological significance of the identified matches.

b IDCP: immunoglobulin domain–containing protein.

**Figure 1.**
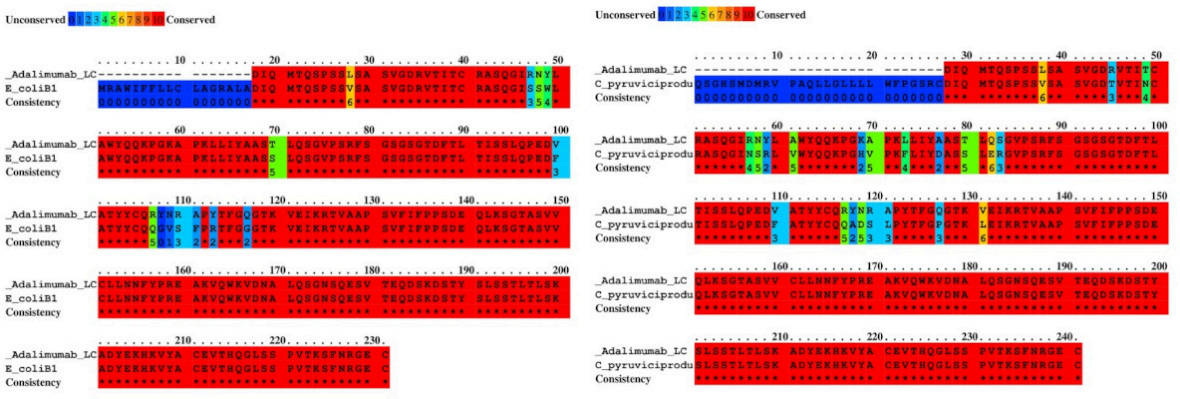
Binary alignment of adalimumab light chain with its closest homologs. The binary alignments demonstrated the highest degree of conservation with adalimumab, *Escherichia coli,* and *Corynebacterium pyruviciproducens*, specifically in residues 18‐231 of *E. coli* and residues 28‐241 of *C. pyruviciproducens.* These findings highlight potential regions of molecular mimicry between adalimumab and bacterial proteins.

To further characterize the similarities detected through PSI-BLASTp, pairwise alignments were conducted between the adalimumab light chain and the bacterial homologs. This analysis was designed to quantify the degree of sequence conservation and to identify bacterial species exhibiting the closest resemblance to the therapeutic antibody. The results showed identity values ranging from moderate to high (43.84%‐94%) between adalimumab and the bacterial antigens. The highest sequence conservation was observed with *E. coli* and *Corynebacterium pyruviciproducens* (Table 1 and Figure 1). These findings suggest that certain bacterial proteins share notable similarity with adalimumab, which may be relevant for understanding potential cross-reactive interactions.

### Modeling and Structural Analysis

**Figure 2.**
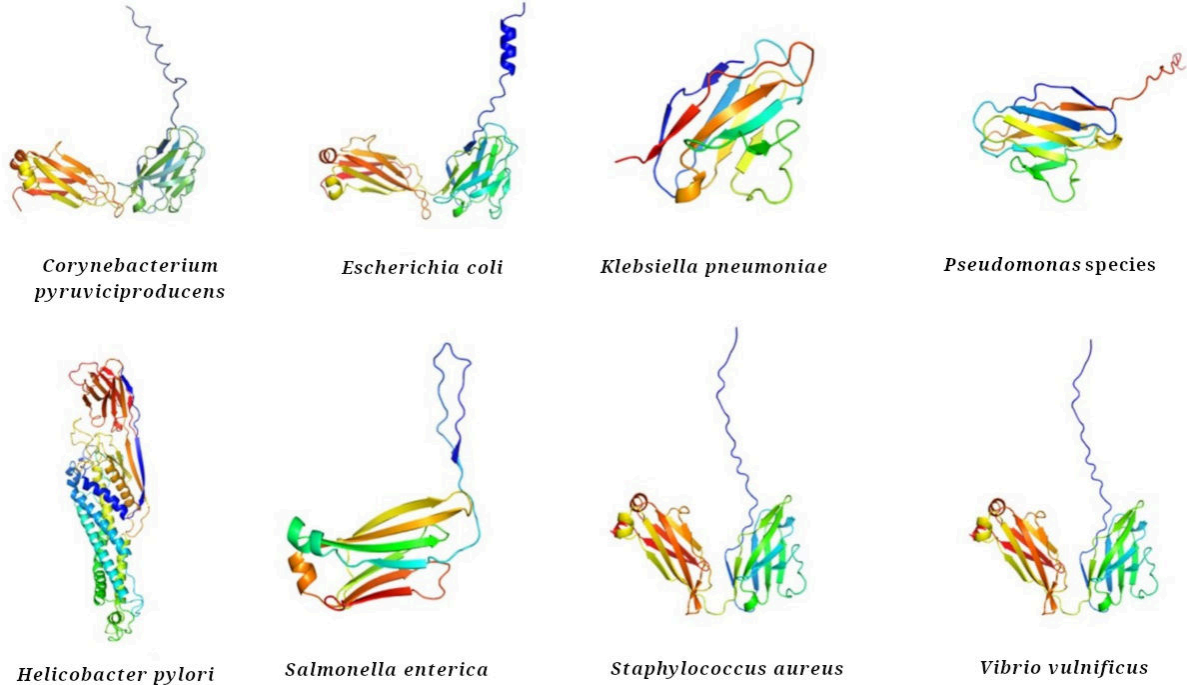
The 3D models. Structures modeled for bacterial antigens homologous to adalimumab light chain adopted a typical fold of an immunoglobulin domain.

**Figure 3.**
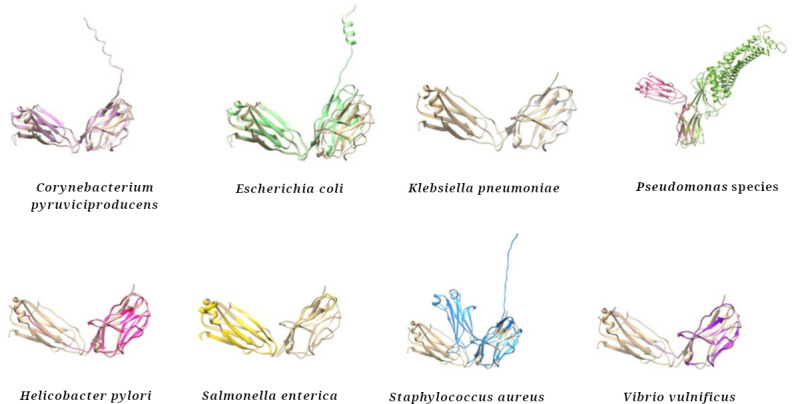
Structural homology. The 3D structure of adalimumab light chain (colored in brown) was superimposed onto each of the modeled structures for bacterial homologs. Analysis revealed a match among the compared structures, indicating a high degree of structural homology.

To evaluate whether the sequence similarities observed translated into comparable 3D conformations, structural models were generated for the bacterial antigens listed in Table 1. This analysis aimed to determine the extent to which these bacterial proteins might adopt folds resembling those of adalimumab, thereby providing structural evidence of potential mimicry. The resulting models showed that the bacterial antigens consistently adopted the characteristic immunoglobulin-like fold (Figure 2). In the cases of *C*. *pyruviciproducens*, *E*. *coli*, *Staphylococcus aureus*, and *Vibrio vulnificus*, the predicted structures were organized as dimers. The GMQE scores indicated reliable model quality, with the lowest value (0.70) corresponding to *Salmonella enterica*. This parameter combines information from the sequence alignment and the quality of the structural template to estimate the expected accuracy of the final model; GMQE values range from 0 to 1, and scores above 0.6 are generally considered indicative of high-confidence structural predictions [26]. Furthermore, RMSD analyses demonstrated a high degree of structural similarity between adalimumab and the bacterial models (Figure 3). RMSD values represent the average atomic distance between 2 superimposed structures, where values below 1 Å suggest a nearly identical spatial organization [36]. In particular, the remarkably low RMSD observed for *E. coli* (0.2 Å) provides compelling evidence of molecular mimicry, as such minimal deviation indicates that both molecules share an almost indistinguishable folding pattern. This structural conservation not only supports the sequence-based similarities but also implies that the bacterial proteins could expose epitopes in a conformation highly compatible with antibody recognition, thereby favoring potential cross-reactivity or recognition from adalimumab antibodies to bacterial proteins and adalimumab.

### B-Cell Epitope Prediction

**Figure 4.**
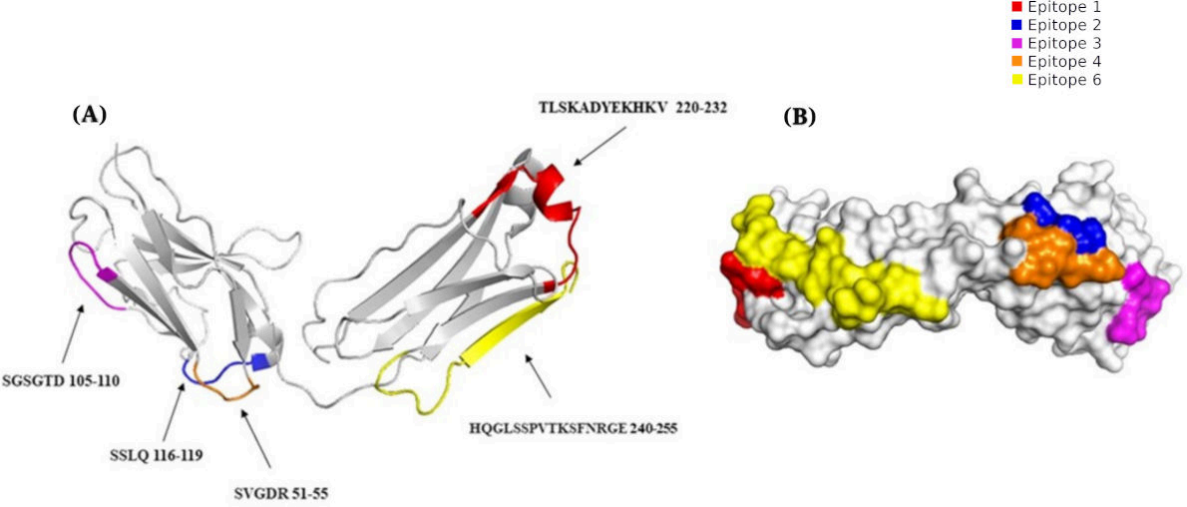
Cross-reactive B-cell epitope prediction. According to Ellipro and multiple alignment tools, 5 linear epitopes are shared between adalimumab light chain and its homologs, which could be implicated in cross-reactivity. (A) A cartoon model illustrates the location of epitopes on the 3D structure of adalimumab. (B) A surface model displays the area occupied by the predicted epitopes.

To explore whether the sequence similarities between adalimumab and bacterial homologs could translate into shared antigenic regions, an in silico prediction of B-cell epitopes was performed. This computational approach aimed to identify potential linear epitopes within adalimumab that might overlap with conserved bacterial sequences and therefore represent possible cross-reactive sites. The analysis predicted 5 B-cell epitopes in adalimumab showing varying degrees of similarity to bacterial homologs (Table 2). The predicted epitopes differed in length, with epitopes 1 and 5 containing the largest number of residues, while epitope 2 comprised only 4 residues shared among the bacterial antigens examined. Structural projection of the predicted epitopes revealed that potential cross-reactive regions are distributed across different areas of the antibody surface (Figure 4A). Additionally, surface modeling indicated that these epitopes collectively occupy a substantial portion of the molecular surface (Figure 4B). Although the predicted epitopes ranged from 4 to 16 amino acids, even short conserved sequences can be relevant for antigen recognition, as complementarity-determining regions within antibodies—often only 6‐20 residues long—are primarily responsible for specific antigen binding [37].

**Table 2.** Epitopes predicted on adalimumab to be conserved among bacterial homologs^a^.

Epitope	Sequences	Start	End	Residues	Score
1	TLSKADYEKHKV	220	232	12	0.802
2	SSLQ	116	119	4	0.5
3	SGSGTD	105	110	6	0.651
4	SVGDR	51	55	5	0.653
5	HQGLSSPVTKSFNRGE	240	255	16	0.676

a The scores assigned to each predicted epitope range from 0 to 1, with values closer to 1 indicating a stronger prediction. A score of 0.802 for epitope 1, for example, suggests a high level of confidence in its conservation and potential functional relevance across bacterial homologs. The biological relevance of these results suggests that, among the entire antigen sequence, the region corresponding to the predicted epitope is the most likely to be immunogenic and therefore to be recognized by antibodies.

### MHC Class II–Dependent T Epitope Prediction

T-cell epitope predictions using the IEDB MHC II binding tool identified multiple adalimumab-derived peptides with strong affinity (percentile ≤2%) for HLA-DRB1 alleles previously associated with anti-adalimumab antibodies. Both heavy- and light-chain regions contained potential CD4^+^ T-cell epitopes presented by these risk alleles, supporting a T-cell–dependent mechanism of immunogenicity (Tables S2 and S3 in Multimedia Appendix 3). Screening of the clinically relevant bacterial proteomes revealed peptides with comparable high-affinity binding to the same HLA molecules and partial sequence similarity to adalimumab epitopes (not shown). This overlap suggests that microbial antigens may share HLA-restricted motifs with adalimumab, potentially enabling cross-reactive T-cell responses that contribute to antidrug antibody formation.

### PC Analysis

PC analysis performed using the IEDB Population Coverage tool showed that the predicted class II epitopes from the adalimumab light chain could be presented by approximately 15.2% of the European population (Multimedia Appendix 4 and Table S4 in Multimedia Appendix 3). The mean number of epitope–HLA combinations recognized per individual was 3.19 (SD ), with a PC90 value of 2.48. These results indicate that a limited yet notable fraction of the population carries HLA alleles capable of presenting these epitopes, supporting the existence of potential interindividual variability in T-cell responsiveness to adalimumab.

### Conservation Analysis of Immunoglobulin Domain in Bacteria

**Figure 5.**
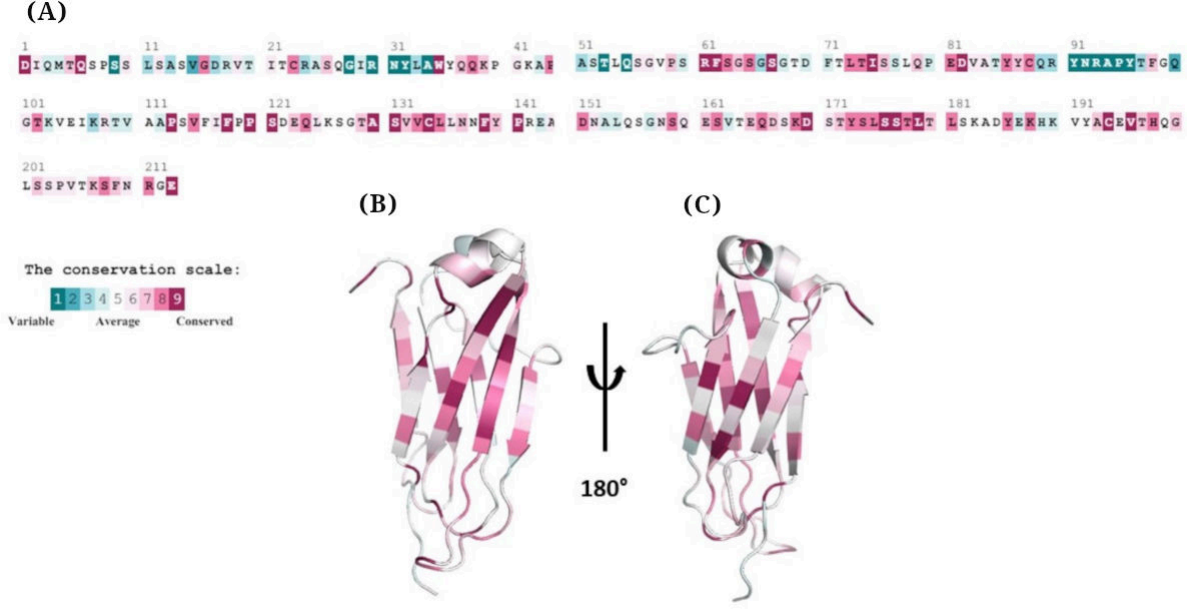
The conservation analysis of individual amino acids in adalimumab was conducted using the ConSurf server. (A) Display of the amino acid sequence, highlighting evolutionarily conserved residues of immunoglobulin domains of the bacterial antigens in adalimumab. (B) and (C) are cartoon models that illustrate the conserved regions on the 3D structure of adalimumab, demonstrating that its amino acid sequence and structure are preserved across different species. The degrees of conservation were mapped onto the sequence and structure, employing the ConSurf color-coding scheme, where shades range from cyan (representing variable, grade 1) to purple (indicating highly conserved, grade 9) positions.

To evaluate whether the structural similarities between adalimumab and bacterial homologs reflect conserved evolutionary features, a conservation analysis of the immunoglobulin domain was performed using the ConSurf server. This analysis aimed to identify amino acid residues that are evolutionarily conserved across bacterial antigens and the antibody, which could indicate the maintenance of structural or functional motifs important for protein stability or interaction. The results, summarized in Figure 5, highlight conserved residues mapped onto the amino acid sequence and the corresponding 3D structures. The cartoon representations illustrate that several regions of the immunoglobulin domain remain highly conserved among the analyzed bacterial species. The conservation score gradient, represented by ConSurf’s color scale from cyan (variable=grade 1) to purple (highly conserved=grade 9), emphasizes that key residues within the core of the domain exhibit strong conservation, suggesting evolutionary pressure to maintain structural integrity in these regions.

### Allergenicity Prediction Using AllerTOP

Allergenicity analysis performed using the AllerTOP server revealed that several of the predicted adalimumab-derived peptides share sequence similarity with known or probable allergens. Notably, matches were identified with allergenic proteins from *Sarcoptes scabiei* (Sarc s 1), *Artemisia vulgaris* (Art v 3), and *Malassezia sympodialis*, as well as with plant-derived allergens such as hydroxyproline-rich glycoproteins from *Oryza sativa* and neoxanthin synthase from *Solanum tuberosum*. Some peptides also showed similarity to human proteins, including ATP synthase F1 assembly factor 2 and B-cell lymphoma 6 protein, suggesting potential immunological cross-reactivity. Overall, the presence of sequences with predicted allergenic properties indicates that these epitopes may elicit immune recognition and could contribute to the immunogenic potential of adalimumab.

## Discussion

### Principal Findings

This study identified several bacterial antigens that share significant sequence and structural similarities with adalimumab, particularly within immunoglobulin-like domains. Using in silico analyses, we found that these bacterial proteins exhibit conserved folds and low RMSD values relative to adalimumab, supporting the hypothesis of molecular mimicry. Such a resemblance may provide a mechanistic explanation for the development of ADAs and the loss of therapeutic response observed in some patients treated with adalimumab. To our knowledge, this is the first study to propose a mechanism of cross-reactivity between adalimumab and microbial antigens supported by in silico structural and sequence evidence. Moreover, it is the first report describing specific bacterial epitopes with potential clinical relevance in the context of adalimumab immunogenicity.

Up to 60% of patients who receive adalimumab eventually show a decline in the effectiveness of the treatment [4]. Therapeutic failure is thought to be caused by ADAs, but the reasons for its formation are still unknown. Some past infections may have contributed to the development of ADAs [15]. In this setting, molecular mimicry could explain why prior infections produce these antibodies. Molecular mimicry may cause antibodies to be generated during past infections to inadvertently neutralize adalimumab by cross-reacting with its epitopes, diminishing its therapeutic effect. So, we propose using bioinformatics to investigate this phenomenon. In support of this hypothesis, our in silico T-cell epitope predictions identified adalimumab peptides with strong HLA-DRB1 binding—alleles previously linked to anti-adalimumab antibodies—and revealed bacterial peptides capable of binding the same HLA molecules, suggesting a potential cross-reactive, T-cell–mediated mechanism underlying ADA formation.

To establish a link between certain microbes and molecular mimicry, we must follow a 4-tiered evidence approach [2,7]. Previously, an epidemiological connection was established [15]. Now, using in silico methods, we have identified potential antigens with significant identity, indicating possible mimicry. Although the analyzed fragments are relatively short, they were selected from clinically relevant bacteria due to their potential involvement in molecular mimicry processes. Information regarding their immunodominant regions is currently unavailable, as this is the first study addressing these specific bacterial-antibody similarities. This finding suggests that clinically relevant bacteria, including *E. coli*, *C. pyruviciproducens*, *Klebsiella pneumoniae*, *V. vulnificus*, *Pseudomonas aeruginosa*, *Helicobacter pylori*, *S. enterica*, and *S. aureus*, may harbor molecular motifs capable of inducing cross-reactivity. These microorganisms are of major medical concern as they encompass commensals with pathogenic potential, such as *E. coli* [38]; opportunistic pathogens linked to severe infections like *C. pyruviciproducens* [39,40]; and multidrug-resistant strains such as *K. pneumoniae* [41,42]. Others, including *V. vulnificus* and *P. aeruginosa*, are associated with life-threatening conditions such as necrotizing soft-tissue infections and ventilator-associated pneumonia, respectively [43,44]. Moreover, *H. pylori*, *S. enterica*, and *S. aureus* contribute to chronic and systemic diseases ranging from gastritis and typhoid fever to osteomyelitis and sepsis [45-47]. In addition, population coverage analysis using the IEDB tool showed that the predicted class II epitopes from the adalimumab light chain could be presented by approximately 15% of the European population, suggesting that only a subset of individuals possess HLA alleles capable of recognizing these epitopes and may therefore be more prone to T-cell–mediated immunogenic responses.

The observed sequence similarity within immunoglobulin-like domains highlights a plausible mechanism through which antibodies targeting microbial antigens could recognize adalimumab epitopes, potentially impairing its therapeutic efficacy. This warrants further experimental validation through in vitro inhibition assays and in vivo studies to confirm the immunological and clinical significance of these mimicry events. These predictions also provide candidate regions for in vitro validation, enabling the refinement of epitope mapping through assays such as enzyme-linked immunosorbent assay or peptide-binding tests. Additionally, the complete amino acid sequences of the bacterial antigens are available for future recombinant cloning and seroprevalence studies in adalimumab-treated patients.

In addition, allergenicity prediction using the AllerTOP server indicated that several adalimumab-derived peptides share sequence similarity with known or probable allergens from diverse sources, including *S. scabiei*, *A. vulgaris*, and *O. sativa*. This overlap suggests that certain epitopes may possess inherent immunostimulatory properties or cross-reactivity with environmental allergens, further supporting their potential contribution to the immunogenic profile of adalimumab.

Our results have cast a new light on the intricate role of molecular mimicry in drug development, merging concepts from diverse therapeutic domains. Historically, molecular structures resembling those of microbial antigens have been exploited to elicit immunological benefits, enhancing the body’s defenses against diseases and improving vaccine efficacy, and even cancer response [48-51]. However, our findings underscore a more intricate reality where these structural analogies, notably those present in adalimumab, bear the potential to induce ADA development. This duality reflects broader pharmaceutical experiences, where beneficial immunogenic mimicry can, in some contexts, inadvertently lead to immune cross-reactions with conditions like thrombocytopenia and infectious and autoimmune diseases [5,10-14]. The resulting immunological disarray, wherein the body cannot differentiate between therapeutic agents and microbial antigens, might trigger an unwarranted immune response against the host’s own tissues, manifesting in a range of adverse clinical outcomes such as autoimmune disease relapses, serum sickness, hypersensitivity reactions, or symptoms of autoimmune disease. Thus, our study bridges the existing knowledge gap by revealing how the same molecular resemblances that have been leveraged for therapeutic gain may also carry risks that must be carefully navigated in the continuum of drug research and patient care.

Unraveling the connections of shared molecular similarities between drugs and various microorganisms—pathogens and commensals alike—can illuminate potential reasons behind the unintended effects some medications might have on the immune system’s response to treatment. Specifically, within the therapeutic framework, molecular mimicry may introduce complexities in treatment modalities due to its potential to elicit aberrant immune responses. Such phenomena can attenuate the efficacy of the medication or precipitate adverse immunological reactions through ADA development. We also consider it may be beneficial to adjust the drug’s formulation and design to lessen its immunogenic potential while maintaining therapeutic effectiveness, a task that is complex yet crucial [52].

In the realm of precision medicine, the significance of molecular mimicry between monoclonal antibodies and microorganisms is profound. This study highlights the presence of antigens with immunoglobulin domains in microbes such as *E. coli*, *H. pylori*, and *S. aureus*, common colonizers [53-55], as well as in those linked to severe diseases, including *K. pneumoniae*, *V. vulnificus*, *S. enterica*, *C. pyruviciproducens*, and *Pseudomonas* species [40,41,43,56,57]. These immunoglobulin domains, which are involved in diverse binding and molecular recognition processes, have been identified across a spectrum of functional groups, including molecular transport, morphoregulation, and cell adhesion to virus receptors, shape recognition, and toxin neutralization [8,9]. The remarkable functional versatility of the immunoglobulin superfamily extends to cell phenotype markers and regulators of gene transcription, among others. Thus, the structural and functional parallels found in this study underscore the need for careful consideration of molecular mimicry in drug development, particularly in the design of monoclonal antibodies, due to their potential to elicit unintended immune responses or interfere with microbial commensals critical to human health.

It is important to acknowledge the limitations of our study. The actual structures may differ from the models we propose, as in silico modeling and epitope prediction analyses are not definitive. Nevertheless, bioinformatic approaches offer significant advantages by efficiently guiding research efforts [27]. They play a crucial role in the initial evaluation of hypotheses, helping determine whether further in vitro studies are warranted. Additionally, bacteria may retain remnants of vectors through horizontal and vertical transmission, whether occurring naturally or during biotechnological processes. However, since not all the bacteria analyzed are associated with biotechnology, any bacterial contamination, if present, is more likely to result from natural processes and evolutionary mechanisms. Moreover, given that all the bacterial antigens studied contain an immunoglobulin-like domain—a feature widely reported across various organisms and particularly in enterobacteria [8]—we consider it unlikely that these findings are due to contamination.

While in silico predictions provide valuable preliminary insights, we acknowledge that they cannot fully confirm the occurrence of molecular mimicry in a biological context. Nonetheless, the results provide a rational basis for the design of forthcoming serological studies, guiding the selection of candidate antigens and regions with potential cross-reactivity. This work represents a first step toward understanding the mechanisms underlying cross-reactive immune responses between microbial antigens and therapeutic antibodies. The high degree of structural similarity observed suggests a biologically plausible mechanism of cross-reactivity, which we plan to further investigate through in vitro validation assays. In addition, we acknowledge that immunogenicity is a multifactorial process influenced not only by molecular mimicry but also by protein-specific properties, manufacturing conditions, and patient-related factors. Nonetheless, molecular mimicry remains one of the least explored contributors to therapeutic antibody immunogenicity, and our findings provide the first evidence-supported hypothesis proposing this mechanism in the context of adalimumab.

As we look toward future pharmaceutical innovation, the insights gained from this inquiry advocate for an integrated approach. This approach should encompass a thorough investigation of the interplay between drugs, the human microbiome, and pathogenic microorganisms. By doing so, we can strive to harness the positive aspects of molecular mimicry while mitigating its risks, thereby advancing the field of medicine with a more informed and cautious perspective.

### Conclusions

In conclusion, examining adalimumab’s structural similarities with key microorganisms such as bacteria offers a nuanced perspective on molecular mimicry’s dual role in medicine. While its utility in enhancing therapeutic benefits is established, we urge a critical reevaluation based on our findings that raise the possibility of adverse immune reactions due to ADAs. Our results also point to the need to advance in the confirmation through in vitro and in vivo tests of this cross-reactivity, because this would make it a necessary and judicious approach to drug design, incorporating an integrated analysis of drug-pathogen-microbiome interactions to safeguard therapeutic efficacy and patient health.

## Supplementary material

10.2196/83872 Multimedia Appendix 1 Computational workflow.

10.2196/83872 Multimedia Appendix 2 Multiple alignment between adalimumab light chain and homologs. The multiple alignments revealed a 64% identity among the compared amino acid sequences. The highest conservation is observed between residues 71 and 159.

10.2196/83872 Multimedia Appendix 3 PSI-BLASTp results and predicted class II T-cell epitopes for adalimumab chains, including HLA-DRB1*11:01–restricted peptides and projected population coverage.

10.2196/83872 Multimedia Appendix 4 Population coverage analysis for class II epitopes predicted from the adalimumab light chain using the Immune Epitope Database (IEDB) population coverage tool. The bar and cumulative distribution plots represent the percentage of individuals in the European population predicted to recognize at least 1 human leukocyte antigen (HLA)–epitope combination, indicating an estimated coverage of approximately 15%.

## Abbreviations

ADA: antidrug antibody
GMQE: Global Model Quality Estimate
HLA: human leukocyte antigen
IDCP: immunoglobulin domain-containing protein
IEDB: Immune Epitope Database
MHC: major histocompatibility complex
PC: population coverage
PDB: Protein Data Bank
PI: protrusion index
RMSD: root mean square deviation
