# Supplementary figures and images for: Immunogenicity of Adalimumab in Bacterial Molecular Mimicry: In Silico Analysis
Source: JMIR Bioinform Biotechnol. 2025 Dec 8;6:e83872. doi: 10.2196/83872 (PMC12685288; doi:10.2196/83872)

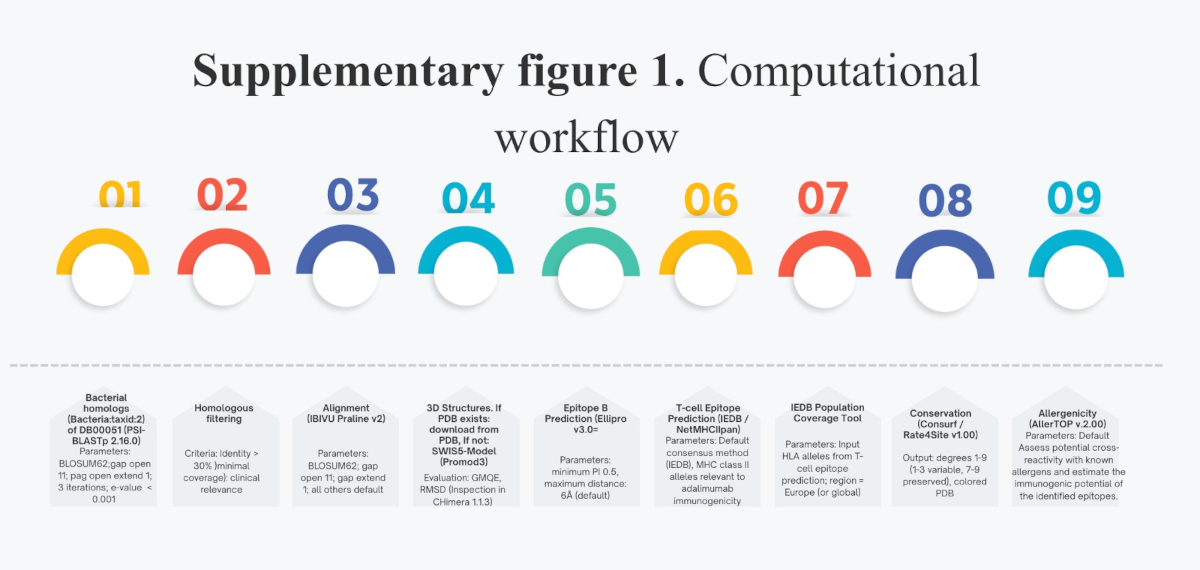

Supplement: Multimedia Appendix 1 [file bioinform-v6-e83872-s001.png]

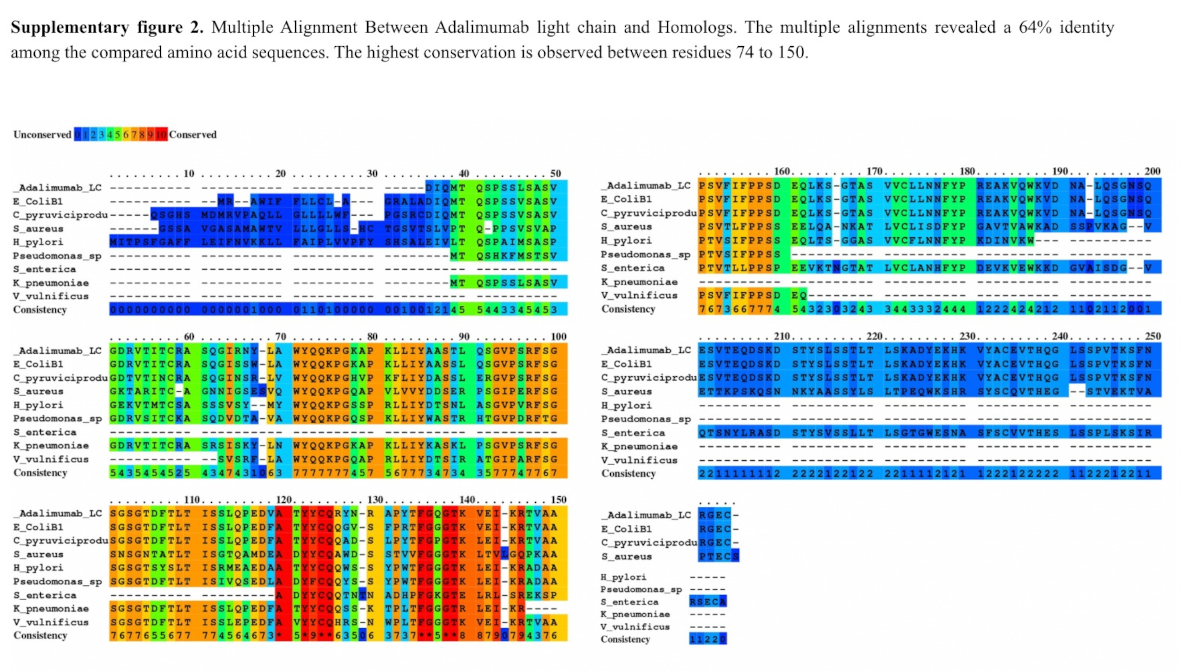

Supplement: Multimedia Appendix 2 [file bioinform-v6-e83872-s002.png]

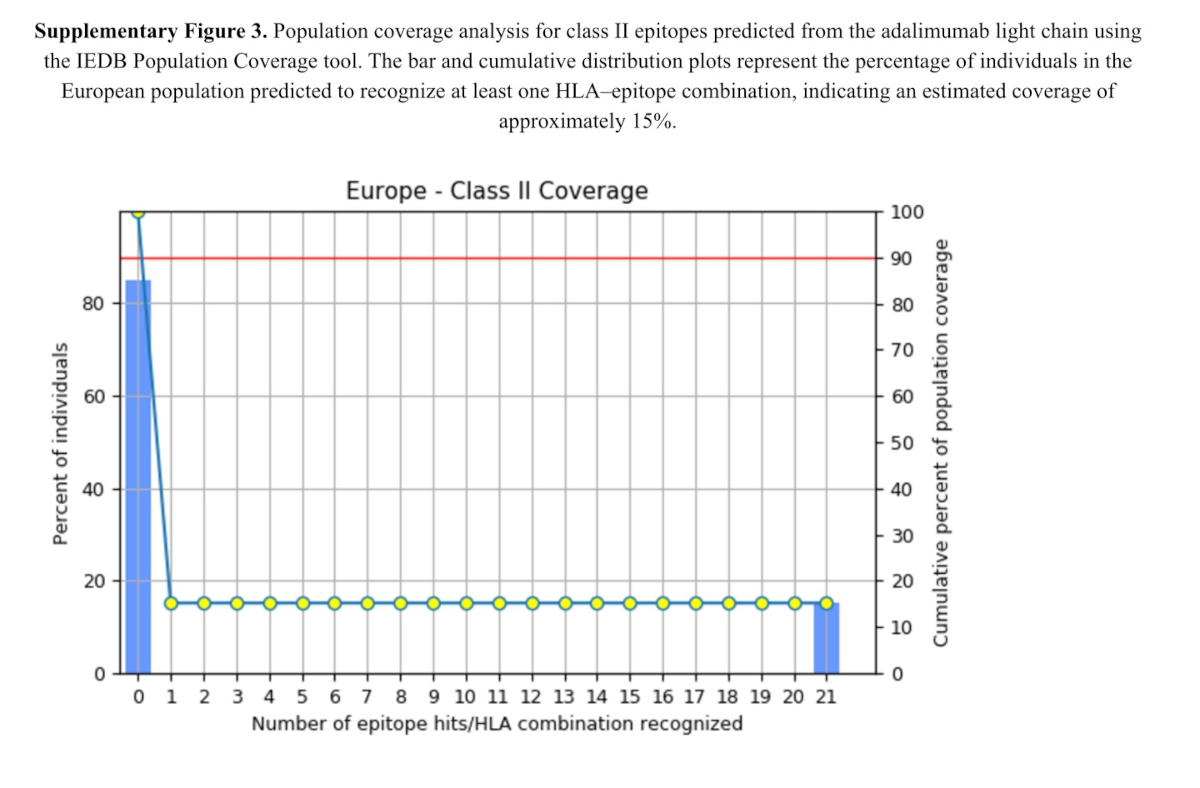

Supplement: Multimedia Appendix 4 [file bioinform-v6-e83872-s004.png]
