# Supplementary material for: Immunogenicity of Adalimumab in Bacterial Molecular Mimicry: In Silico Analysis
Source: JMIR Bioinform Biotechnol. 2025 Dec 8;6:e83872. doi: 10.2196/83872 (PMC12685288; doi:10.2196/83872)
Supplement: Multimedia Appendix 3 [file bioinform-v6-e83872-s003.docx]

**Table S1**

**PSI-BLASTp results for the heavy chain of adalimumab.** This table presents the structural similarity between bacterial homologs and adalimumab heavy chain thorugh PSI-BLASTp analysis.

| **Bacteria** | **Antigen** | **Similarity** | **Cod Genbank** |
| --- | --- | --- | --- |
| *Pseudomonas aeruginosa* | Hypothetical protein, partial | 87% | WP_270036806.1 |
| *Escherichia coli* | IDCP, partial | 82% | MCL7168995.1 |
| *Klebsiella pneumoniae* | Hypothetical protein, partial | 70.94% | WP_142519433.1 |
| *Helicobacter pylori* | IDCP, partial | 64.29% | WP_304481311.1 |
| *Salmonella enterica* | Hypothetical protein, partial | 57.76% | MCQ7618865.1 |
| *Staphylococcus aureus* | Hypothetical protein, partial | 52.71% | WP_181935871.1 |
| *Vibrio vulnificus* | Hypothetical protein, partial | 31% | WP_349966564.1 |
| *Corynebacterium pyruviciproducens* | IDCP, partial | 27.2% | WP_280195946.1 |

**IDCP:**Immunoglobulin domain-containing protein.

**Table S2.** Predicted class II T-cell epitopes from the adalimumab light chain obtained using the IEDB MHC II binding tool. Several high-affinity peptides, particularly for HLA-DRB1*11:01, were identified, indicating potential regions involved in CD4⁺ T-cell recognition and adalimumab immunogenicity.

| **Allele** | **Seq num** | **Start** | **End** | **Length** | **Core peptide** | **Peptide** | **Score** | **Rank** |
| --- | --- | --- | --- | --- | --- | --- | --- | --- |
| HLA-DRB1*11:01 | 4 | 9 | 23 | 15 | YACEVTHQG | HKVYACEVTHQGLSS | 0.5536 | 2.9 |
| HLA-DRB1*11:01 | 3 | 25 | 39 | 15 | WKVDNALQS | KVQWKVDNALQSGNS | 0.5497 | 2.9 |
| HLA-DRB1*11:01 | 4 | 8 | 22 | 15 | YACEVTHQG | KHKVYACEVTHQGLS | 0.5003 | 3.4 |
| HLA-DRB1*11:01 | 2 | 41 | 55 | 15 | VEIKRTVAA | GTKVEIKRTVAAPSV | 0.4917 | 3.6 |
| HLA-DRB1*11:01 | 3 | 24 | 38 | 15 | WKVDNALQS | AKVQWKVDNALQSGN | 0.4732 | 3.8 |
| HLA-DRB1*11:01 | 1 | 16 | 30 | 15 | VTITCRASQ | GDRVTITCRASQGIR | 0.4614 | 3.9 |
| HLA-DRB1*11:01 | 2 | 40 | 54 | 15 | VEIKRTVAA | QGTKVEIKRTVAAPS | 0.4501 | 4.1 |
| HLA-DRB1*11:01 | 2 | 8 | 22 | 15 | FTLTISSLQ | GTDFTLTISSLQPED | 0.3569 | 5.4 |
| HLA-DRB1*11:01 | 4 | 7 | 21 | 15 | YACEVTHQG | EKHKVYACEVTHQGL | 0.3284 | 5.9 |
| HLA-DRB1*11:01 | 2 | 39 | 53 | 15 | VEIKRTVAA | GQGTKVEIKRTVAAP | 0.3064 | 6.2 |
| HLA-DRB1*11:01 | 1 | 45 | 59 | 15 | IYAASTLQS | KLLIYAASTLQSGVP | 0.3038 | 6.3 |
| HLA-DRB1*11:01 | 2 | 42 | 56 | 15 | IKRTVAAPS | TKVEIKRTVAAPSVF | 0.2992 | 6.4 |
| HLA-DRB1*11:01 | 4 | 10 | 24 | 15 | YACEVTHQG | KVYACEVTHQGLSSP | 0.2925 | 6.6 |
| HLA-DRB1*11:01 | 3 | 16 | 30 | 15 | FYPREAKVQ | LNNFYPREAKVQWKV | 0.2906 | 6.6 |
| HLA-DRB1*11:01 | 1 | 15 | 29 | 15 | VTITCRASQ | VGDRVTITCRASQGI | 0.2862 | 6.7 |
| HLA-DRB1*11:01 | 3 | 23 | 37 | 15 | WKVDNALQS | EAKVQWKVDNALQSG | 0.2781 | 6.8 |
| HLA-DRB1*11:01 | 3 | 15 | 29 | 15 | FYPREAKVQ | LLNNFYPREAKVQWK | 0.2634 | 7.2 |
| HLA-DRB1*11:01 | 3 | 26 | 40 | 15 | WKVDNALQS | VQWKVDNALQSGNSQ | 0.2291 | 8.1 |
| HLA-DRB1*11:01 | 2 | 43 | 57 | 15 | IKRTVAAPS | KVEIKRTVAAPSVFI | 0.221 | 8.4 |
| HLA-DQA1*01:01/DQB1*03:01 | 3 | 31 | 45 | 15 | LQSGNSQES | DNALQSGNSQESVTE | 0.218 | 0.3 |

**Table S3.** Predicted class II T-cell epitopes from the adalimumab heavy chain obtained using the IEDB MHC II binding tool. Several high-affinity peptides, mainly restricted by HLA-DRB1*11:01, were identified, suggesting possible CD4⁺ T-cell recognition sites involved in adalimumab immunogenicity

| **Allele** | **Seq num** | **Start** | **End** | **Length** | **Core peptide** | **Peptide** | **Score** | **Rank** |
| --- | --- | --- | --- | --- | --- | --- | --- | --- |
| HLA-DRB1*11:01 | 2 | 17 | 31 | 15 | YLQMNSLRA | NSLYLQMNSLRAEDT | 0.7682 | 1.2 |
| HLA-DRB1*11:01 | 6 | 20 | 34 | 15 | YKCKVSNKA | GKEYKCKVSNKALPA | 0.7511 | 1.3 |
| HLA-DRB1*11:01 | 2 | 16 | 30 | 15 | YLQMNSLRA | KNSLYLQMNSLRAED | 0.7203 | 1.6 |
| HLA-DRB1*11:01 | 6 | 19 | 33 | 15 | YKCKVSNKA | NGKEYKCKVSNKALP | 0.6922 | 1.8 |
| HLA-DRB1*11:01 | 6 | 18 | 32 | 15 | YKCKVSNKA | LNGKEYKCKVSNKAL | 0.5085 | 3.3 |
| HLA-DRB1*11:01 | 7 | 45 | 59 | 15 | FFLYSKLTV | DGSFFLYSKLTVDKS | 0.4969 | 3.5 |
| HLA-DRB1*11:01 | 6 | 21 | 35 | 15 | YKCKVSNKA | KEYKCKVSNKALPAP | 0.4914 | 3.6 |
| HLA-DRB1*11:01 | 2 | 15 | 29 | 15 | YLQMNSLRA | AKNSLYLQMNSLRAE | 0.435 | 4.3 |
| HLA-DRB1*11:01 | 7 | 44 | 58 | 15 | FFLYSKLTV | SDGSFFLYSKLTVDK | 0.4136 | 4.6 |
| HLA-DRB1*11:01 | 2 | 18 | 32 | 15 | YLQMNSLRA | SLYLQMNSLRAEDTA | 0.3985 | 4.8 |
| HLA-DRB1*11:01 | 3 | 9 | 23 | 15 | LAPSSKSTS | VFPLAPSSKSTSGGT | 0.3563 | 5.4 |
| HLA-DRB1*11:01 | 8 | 17 | 31 | 15 | YTQKSLSLS | HNHYTQKSLSLSPGK | 0.3491 | 5.5 |
| HLA-DRB1*11:01 | 6 | 1 | 15 | 15 | YRVVSVLTV | NSTYRVVSVLTVLHQ | 0.3053 | 6.3 |
| HLA-DRB1*11:01 | 3 | 8 | 22 | 15 | LAPSSKSTS | SVFPLAPSSKSTSGG | 0.2836 | 6.7 |
| HLA-DRB1*11:01 | 4 | 19 | 33 | 15 | YICNVNHKP | TQTYICNVNHKPSNT | 0.2584 | 7.3 |
| HLA-DRB1*11:01 | 7 | 32 | 46 | 15 | YKTTPPVLD | ENNYKTTPPVLDSDG | 0.2471 | 7.6 |
| HLA-DRB1*11:01 | 8 | 16 | 30 | 15 | YTQKSLSLS | LHNHYTQKSLSLSPG | 0.2377 | 7.9 |
| HLA-DRB1*11:01 | 7 | 46 | 60 | 15 | FFLYSKLTV | GSFFLYSKLTVDKSR | 0.2304 | 8.1 |
| HLA-DRB1*11:01 | 6 | 34 | 48 | 15 | EKTISKAKG | APIEKTISKAKGQPR | 0.2193 | 8.5 |
| HLA-DRB1*11:01 | 1 | 29 | 43 | 15 | YAMHWVRQA | FDDYAMHWVRQAPGK | 0.2168 | 8.6 |
| HLA-DRB1*11:01 | 4 | 18 | 32 | 15 | YICNVNHKP | GTQTYICNVNHKPSN | 0.2063 | 8.9 |
| HLA-DRB1*11:01 | 6 | 17 | 31 | 15 | YKCKVSNKA | WLNGKEYKCKVSNKA | 0.2058 | 8.9 |
| HLA-DQA1*01:01/DQB1*03:01 | 1 | 3 | 17 | 15 | SGGGLVQPG | QLVESGGGLVQPGRS | 0.2008 | 0.44 |

**Table S4.** Projected population coverage for class II epitopes from the adalimumab light chain showing an estimated 15.2% coverage in the European population, with an average of 3.19 epitope–HLA hits per individual (pc90 = 2.48).

| **population/area** | **Class II** | | |
| --- | --- | --- | --- |
|  | **coveragea** | **average_hitb** | **pc90c** |
| [Europe](https://tools.iedb.org/population/result/#Europe) | 15.19% | 3.19 | 2.48 |
|  |  |  |  |
| **Average** | **15.19** | **3.19** | **2.48** |
| **Standard deviation** | **0.0** | **0.0** | **0.0** |

a projected population coverage

b average number of epitope hits / HLA combinations recognized by the population

c minimum number of epitope hits / HLA combinations recognized by 90% of the population
